# Nighttime Pistachio Consumption Alters Stool Microbiota Diversity and Taxa Abundance Compared with Education to Consume 1–2 Carbohydrate Exchanges (15–30 grams) over 12 Weeks in Adults with Prediabetes: A Secondary Analysis from a Randomized Crossover Trial

**DOI:** 10.1016/j.cdnut.2025.107481

**Published:** 2025-05-27

**Authors:** Terrence M Riley, Justin Wright, Regina Lamendella, Jordan E Bisanz, Jeremy Chen See, Khushi Kanani, Penny M Kris-Etherton, Kristina S Petersen

**Affiliations:** 1 Pennington Biomedical Research Center, Louisiana State University, Baton Rouge, LA, United States; 2 Department of Nutritional Sciences, Pennsylvania State University, University Park, PA, United States; 3 Wright Labs, LLC, Huntingdon, PA, United States; 4 Department of Biology, Juniata College, Huntingdon, PA, United States; 5 Department of Biochemistry and Molecular Biology, Pennsylvania State University, University Park, PA, United States

**Keywords:** pistachio, nuts, diet, diet quality, carbohydrate exchanges, 16s rRNA sequencing, glycemic control

## Abstract

**Background:**

Prior research has demonstrated that pistachio intake influences gut microbiota composition; however, there has been limited investigation of pistachio-related gut microbial modulation in populations with impaired glycemia.

**Objectives:**

The aim was to examine the effect of nighttime pistachio intake for 12 wk on the stool microbiota of adults with prediabetes [fasting plasma glucose (FPG) ≥100 mg/dL and ≤125 mg/dL].

**Methods:**

A secondary analysis of data from a single-blind, 2-period, randomized crossover trial was conducted. Participants were provided with 57 g/d of dry roasted unsalted pistachios to consume as a nighttime snack or provided education to consume 1–2 carbohydrate (CHO; 15–30 g) exchanges (usual care) as a nighttime snack for 12 wk. Stool samples were collected at the beginning and the end of each condition and analyzed with 16S ribosomal ribonucleic acid gene sequencing. Taxonomic diversity was tested with linear mixed models (LMMs) and permutational analysis of variance of Bray–Curtis and weighted Unifrac dissimilarity indices. Taxonomic abundance by condition was tested using LMMs with Benjamini–Hochberg multiple testing correction.

**Results:**

The analytical sample included 51 participants (37% female, 49 ± 10 y, 31.5 ± 4 kg/m^2^, FPG 102 ± 10 mg/dL) who completed the trial (of 66 randomly assigned). β-diversity analysis showed community dissimilarity between the pistachio and usual care conditions postcondition (*P* = 0.001). Compared with the usual care condition, nighttime pistachio consumption modified several microbial taxa.

**Conclusions:**

In adults with prediabetes, intake of 57 g/d of pistachios as a nighttime snack altered stool microbial community diversity and composition compared with a CHO-rich snack, providing evidence of stool microbial effects with pistachio consumption.

**Trial registration number:**

This study was registered at clinicaltrials.gov as NCT04056208.

## Introduction

The human gut microbiota is shaped predominantly by nongenetic factors, of which diet is a key short- and long-term regulator [1]. Nuts are a recommended component of healthful dietary patterns [2] that beneficially modulate the gut microbiota across the lifespan [3]. Compared with other commonly consumed nuts, pistachios are rich in fiber, essential amino acids, potassium, choline, vitamins A and K, phytosterols, and phenolic compounds [4]. These components may act as substrates shaping the composition and function of the gut microbiota [5]. Gut compositional properties are an increasingly recognized factor in chronic disease development, partly because they are indicators of microbiota participation in host metabolism, including energy supply to colonocytes, immune response signaling, vitamin synthesis, and pathogen protection [6]. Characterizing how pistachio consumption influences the gut microbiota may further our understanding of the role of pistachios in health promotion and disease prevention.

Pistachio consumption has been shown to modulate the gut microbial composition. Generally, healthy adults consuming 42.5 g/d or 85 g/d of pistachios for 18 d had a shift in bacterial community diversity driven by greater abundance of butyrate-producing bacteria [7]. A greater capacity for butyrate production is linked to improved postprandial insulin response [8] and reductions in glycated hemoglobin (HbA1c) [9], suggesting that pistachios may benefit those with impaired glucose metabolism. Although pistachios may have glucomodulatory effects among those with prediabetes, type 2 diabetes mellitus (T2DM), and cardiovascular disease risk factors [10,11], limited work has described the microbial response to pistachio consumption in these populations. A study among adults with prediabetes showed that daily pistachio consumption (57 g/d) reduced urine concentrations of hippurate, a microbiota-related metabolite positively associated with insulin resistance and T2DM risk [12]. However, the microbial composition was not reported, so it is unclear if compositional changes accompanied these metabolic benefits. Given that diet is a major component of management and treatment strategies for prediabetes and T2DM [13], identifying the microbial effects of pistachio consumption among those with prediabetes may help to understand the role of pistachios in promoting gut and metabolic health.

The objective of this prespecified secondary analysis of stool samples from a previously conducted crossover trial in adults with prediabetes [14] was to evaluate the compositional changes in the microbiota when comparing nighttime pistachio consumption (57 g/d) with education to consume 1–2 carbohydrate (CHO) exchanges (usual care) for 12 wk. The usual care condition reflects common clinical guidance to address morning elevated FPG [15] by providing education to identify foods with 15 g of CHOs (1 exchange) and consume 15–30 g of CHO as a nighttime snack. In the primary publication, we reported that there were no between-condition differences in glycemic outcomes [e.g., FPG (primary outcome), HbA1c, insulin, HOMA-IR], lipids/lipoproteins, blood pressure, or vascular health, but healthy eating index (HEI)-2015 scores were higher after the pistachio condition [6.8 points (95% confidence interval (CI): 1.5, 12.1)] than those after the usual care condition [14]. In this article, we report on gut microbiota composition assessed by 16S ribosomal ribonucleic acid (16S rRNA) gene sequencing to compare alpha (⍺)- and beta (β)-diversity and relative taxa abundance [16,17]. We hypothesized that pistachio consumption would promote beneficial shifts in microbial diversity when compared with the usual care condition.

## Methods

### Study design

Details of the trial design and results from the primary outcome and secondary cardiometabolic and dietary outcomes are reported elsewhere [14]. Here, we report results from a planned secondary analysis assessing stool bacterial composition and diversity, including only participants who completed the study. Briefly, a single-blind, 2-period, randomized crossover trial among adults with prediabetes (FPG 100 mg/dL ≤125 mg/dL) was conducted. Participants received the following 2 conditions in random order for 12-wk with at least a 4-wk break between conditions: *1*) provision of 57 g/d of pistachios with instructions to consume them as a nighttime snack; *2*) education to consume 1–2 CHO exchanges as a nighttime snack (usual care). Participants were asked to consume the foods after dinner but before bedtime, and have no other energy-containing foods or beverages after the nighttime snack. In addition, participants were asked to avoid peanuts and tree nuts throughout the trial. Overall adherence to the intervention was high (93% during the usual care and 90% during the pistachio condition). This study was approved by the Pennsylvania State University Institutional Review Board. Written informed consent was obtained from all participants prior to study screening. This study is registered at clinicaltrials.gov identifier NCT04056208.

### Participants

Participants were recruited from State College, Pennsylvania and surrounding areas from September 2019 to September 2023. Individuals interested in participating were screened by telephone to determine eligibility. Eligible individuals attended a follow-up clinic screening visit to determine study eligibility. Males and females aged 30–65 y with a BMI (in kg/m^2^) ≥25 and ≤45 and elevated FPG (≥100 mg/dL and ≤125 mg/dL) measured at screening were eligible. The exclusion criteria included a diabetes diagnosis (any type); systolic blood pressure >160 mmHg or diastolic blood pressure >100 mmHg; use of antihypertensive, lipid-lowering or glucose-lowering drugs; use of steroids or antibiotics in the previous month.

### Dietary assessment

Dietary intake was assessed by nonrandom, participant-completed 24-h recalls prior to each condition and in the last week of each diet period (a total of four 24-h recalls during the study). The Automated Self-Administered 24-Hour Dietary Assessment Tool (ASA24) [18] was used to obtain the 24-h recalls. The healthy eating index (HEI)-2020 was used to assess diet quality [19]. The HEI-2020 was calculated using the Statistical Analysis System (SAS) code created by the National Cancer Institute (NCI) [20]. Recalls were excluded if reported energy intake was <600 or >4400 kcal/d for females and <650 or >5700 kcal/d for males. These cutoffs were derived from the NCI guidelines for reviewing and cleaning ASA24 data [21].

### Stool sample collection

Stool samples were collected from a single defecation prior to each condition and at the end of each condition. Participants could collect the sample at any time throughout the day. Samples were collected within 48 h prior to testing at each timepoint. Participants were provided with a collection kit (Ziploc bags, cooler, icepack, nitrile gloves, a long-handled spoon, a stool collection hat, and 2 × 30 mL Para-Pak Clean Vials; Meridian Bioscience). Participants were instructed to store stool samples in their freezer (<48-h prior to study visit) and transport the samples to the Clinical Research Center using the provided cooler and ice packs. On arrival, samples were stored at –80°C until analysis. All 16S rRNA analyses were completed at Wright Labs, LLC.

### DNA extraction and quantification

DNA was extracted from samples using the Zymo Research ZymoBIOMICS DNA/RNA Mini Kit (Zymo Research Corporation). DNA was eluted using 50 μL of DNase/RNase-free water and quantified using an Invitrogen Qubit 4 Fluorometer and 1X Qubit dsDNA High Sensitivity Assay Kit (Thermo Fisher Scientific,) after extraction.

All 16S rRNA Illumina-tag PCR was performed on DNA extracts as described by the Earth Microbiome Project's protocol [22]. PCR products were pooled, and quality checked using an Agilent 2100 BioAnalyzer and Agilent DNA High Sensitivity DNA kit (Agilent Technologies). The purified pool was stored at –20°C after gel purification on a 2% agarose gel with the QIAquick Gel Purification Kit (Qiagen). The purified pool was sequenced at Wright Labs, LLC, using an Illumina MiSeq v2 chemistry with paired-end 250 base pair reads.

### Bioinformatic analysis procedures

Raw data were imported into Quantitative Insights Into Microbial Ecology 2 (QIIME2) for processing and analyses [23]. Initial quality indicated by Phred q scores were determined using QIIME2, and the cumulative expected error for each position was determined with VSEARCH [23]. Using these quality data, forward reads were truncated at a base length of 170, and reverse reads were truncated at a base length of 150. The maximum expected error was 0.5 for both within QIIME2’s implementation of the DADA2 pipeline [24]. QIIME2’s DADA2 pipeline was also used to merge forward and reverse reads, remove chimeras, and assign the remaining sequences to amplicon sequence variants (ASVs).

Representative sequences were used to determine taxonomic information for the ASVs, using a Naive Bayes classifier as implemented in QIIME2’s “qiime feature-classifier classify-sklearn” command, with a pretrained Silva 138 database containing 515F/806R sequences [25]. Representative sequences were also used to create a rooted phylogenetic tree using Multiple Alignment using Fast Fourier Transform and FastTree through QIIME2’s “qiime phylogeny align-to-tree-mafft-fasttree” command [26,27].

ASVs identified as mitochondria or chloroplasts were removed on the basis that they likely represented eukaryotic contamination rather than a true bacterial signal. Samples with <1000 sequences remaining after that filtration were removed from the ASV table.

### Statistical analysis

The study was originally powered for the primary outcome, FPG [14]. Power calculations were not done for the outcomes reported in this article.

#### α-Diversity analysis

Alpha diversity was assessed by 6 methods. Faith’s Phylogenetic Diversity was calculated by subsampling the ASV table at 10 different depths [28]. Counts of observed ASVs [29], Pielou’s Evenness Index [30], Chao-1 Index [31], Shannon Index [32], and Simpson Index [33] were calculated after rarefying. Averages were calculated with a custom R function that randomly subsampled the abundance table (rrarefy) and calculated ⍺-diversity metrics after each subsampling of the raw values. This was repeated 100 times to ensure robust estimation of diversity measures. Rarefying to the minimum sequencing depth (counts = 10,000) was performed using the vegan package (v2.6-4) [34] to mitigate differences between samples based on sequencing depth [35]. Results from the subsampling were used to create a rarefaction plot and confirm that diversity approached an asymptote and slope decreased as depth increased (data not reported).

The α-diversity analyses were conducted using R Studio software (version 4.3.2). Normality of residuals was assessed for each metric based on the distribution and normal probability plots (Q-Q plots). Metrics were transformed (square root) in the instance of skewed residuals. Linear mixed models (LMMs; R package lme4, v.1.1-35.5) were used to assess between-condition effects on each of the 6 ⍺-diversity metrics [36]. Precondition values for each outcome were included as a covariate. Participant nested within condition was included as a random effect in the model to account for the crossover design (base model: postcondition value = condition + precondition value + participant). Carryover effects and sex differences were determined by including randomization sequence, sex, and their interaction by condition (i.e., sex∗condition, randomization sequence∗condition) in the model as fixed effects. Nonsignificant sex and randomization sequence effects were removed from the final model.

#### β-Diversity analysis

β-diversity was determined using Bray–Curtis dissimilarity with rarefaction (avgdist function, *n* = 100 repeats) and with weighted Unifrac dissimilarity [37]. Weighted Unifrac metrics were based on the normalized table and rooted tree. A principal coordinates analysis plot was used to visualize the resulting distance matrices. Bray–Curtis dissimilarity was also plotted with nonmetric dimensional scaling (NMDS) to account for heterogeneity not captured by metric-dimensional scaling. The adonis2 function in the vegan R package was used to perform permutational analysis of variance (PERMANOVA; 999 permutations) to determine community dissimilarity by timepoint with participant included as a strata variable to account for within-participant effects. To assess between-condition differences in microbial community while accounting for precondition values, a LMM for each axis was used. The model included distance values along each principal coordinate transformed using the functions cmdscale and metaMDS. Postcondition distance values were included in the model as the dependent variable. Condition and precondition values were the fixed independent variables and participant was a random independent variable. Testing between sample community (β-) diversity by axis also allowed for the inclusion of sex and carryover effects where randomization sequence, sex, and their interaction by condition (i.e., sex∗condition, randomization sequence∗condition) were added as fixed effects. The diet quality score was included as a covariate to determine the contribution to variance in microbial community dissimilarity. Reported values from LMMs are postcondition least-squares mean differences with 95% CIs.

#### Taxonomic comparisons

Differential abundance of microbial taxa was assessed using the taxonomy to the genus and species level where applicable. Raw microbial taxa counts were filtered by removing taxa not present in <2 samples with a total of <2 reads. The filtered table was normalized using the R package qiime2R that transforms the data using a centered log2-ratio [38]. Zeros were handled using the count zero multiplicative method. Transformed counts for each microbial feature were tested in an LMM using the lme4 package (v.1.1-35.1). The transformed microbial feature abundance postcondition was set as the dependent variable, condition and precondition microbial feature as fixed independent variables, and participant as a random independent variable. The Benjamini–Hochberg method was used to correct for false discovery from multiple testing with a *P* < 0.1 significance threshold. Reported values from LMMs are least-squares mean differences in relative sample abundance between conditions with 95% CIs.

## Results

### Analytical sample

A total of 66 participants were randomly assigned and 51 participants completed the study. Reasons for noncompletion have been previously described but included loss to follow-up because of study suspension during the COVID-19 pandemic as the most common reason [14]. The analytical sample for these analyses includes the 51 participants who completed the trial. Participant characteristics are presented in Table 1. Nutrient intake by condition and timepoint is presented in Supplemental Table 1.

**TABLE 1:** Characteristics of study completers overall and by randomization sequence at baseline of diet period 1 (*n* = 51)^1^.

Characteristic	Pistachio→usual care	Usual care→pistachio	Total
*n* (% female)	25 (32)	26 (50)	51 (37.2)
Age (y)	49.8 (11.0)	49.6 (10.7)	49.7 (10.7)
Weight (kg)	93.2 (13.0)	94.8 (18.9)	94.0 (16.2)
Height (m)	1.72 (0.08)	1.72 (0.09)	1.73 (0.09)
BMI (kg/m^2^)	31.2 (3.6)	31.7 (4.8)	31.5 (4.2)
FPG (mg/dL)	103.0 (13.3)	101.5 (7.2)	102.3 (10.6)
Insulin (μU/mL)	14.3 (12.0)	12.8 (7.6)	13.6 (9.9)
HOMA-IR	4.8 (8.6)	3.5 (2.3)	4.2 (6.2)
HbA1c (%)	5.5 (0.3)	5.4 (0.3)	5.5 (0.3)
HEI-2020 score	53.3 (13.7)	51.0 (15.6)	52.2 (14.5)

Abbreviations: BMI, body mass index; FPG, fasting plasma glucose; HbA1c, glycated hemoglobin; HEI-2020, healthy eating index-2020; HOMA-IR, Homeostatic Model Assessment of Insulin Resistance.

1 Data are mean (SD) unless otherwise stated. Usual care is defined as education to consume 1–2 carbohydrate (CHO) exchanges each night (15–30 g CHO).

Stool samples were available for 50 participants at all timepoints; 1 participant provided stool samples at 3 of the timepoints. 16S rRNA sequencing was completed on all available samples (*n* = 203). 16S rRNA gene PCR amplification of the hypervariable V4 region was successfully completed on all samples*.* High-quality sequencing data (≥1000 sequences with maximum expected error <0.5) were obtained from 199 stool samples containing 5,816,199 total read counts.

### ⍺-Diversity

**FIGURE 1:**
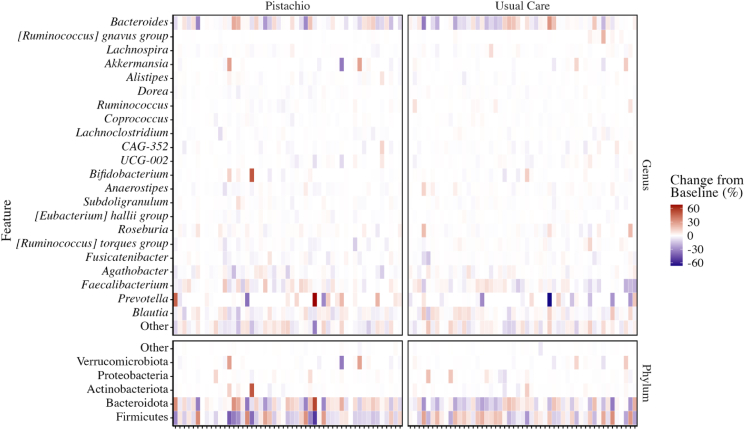
Change in relative abundance of microbial taxa from precondition abundance for each participant. Relative abundance is expressed as the proportion of counts for each microbial feature in a sample divided by the total number of counts observed in the sample at the phyla and [genus] or species levels. Taxa categorized as “other” were on average <1% of observed taxa. Individual participants are on the *x*-axis.

**FIGURE 2:**
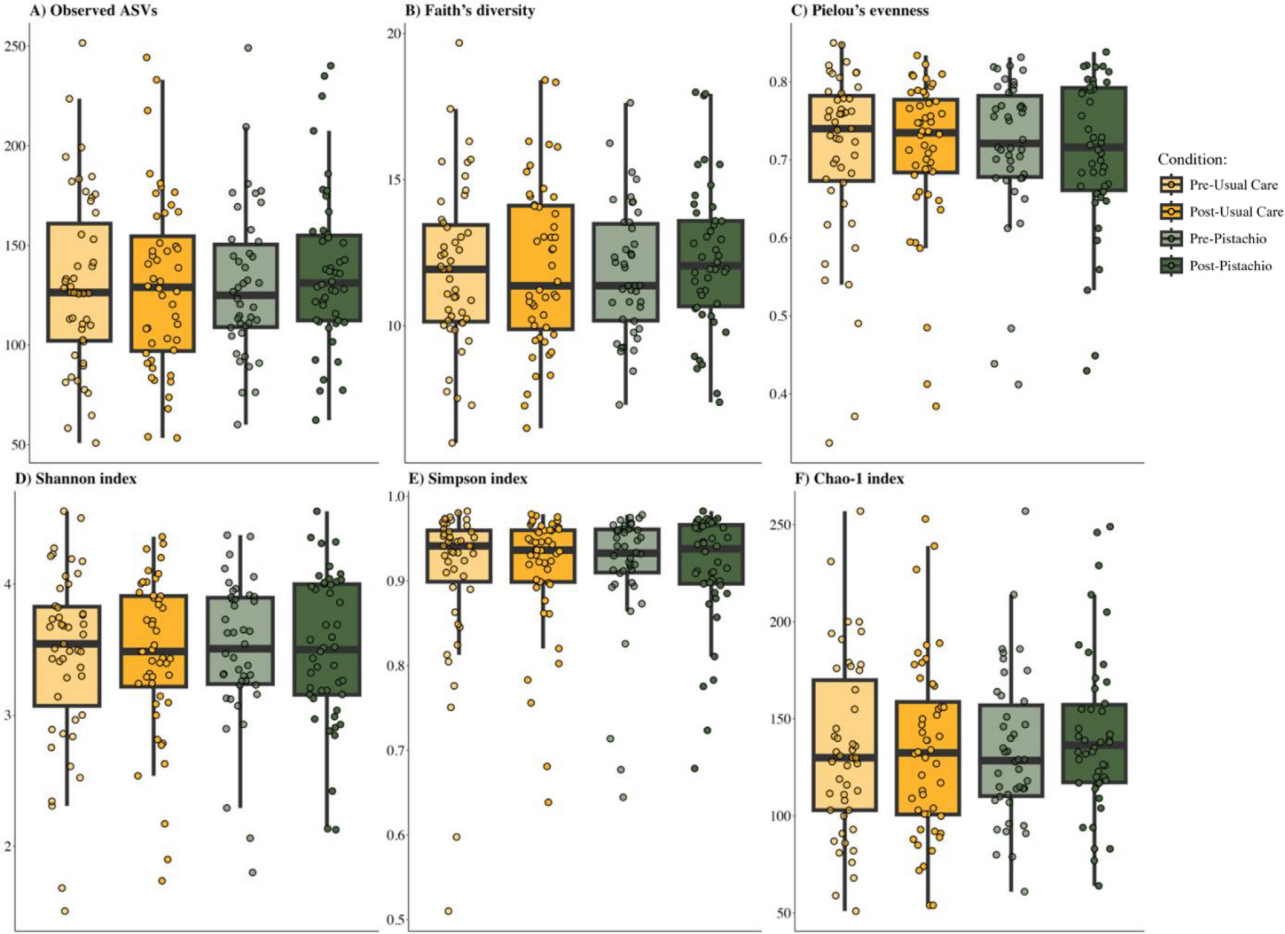
Microbial community α-diversity at each timepoint. α-diversity metrics are expressed as (A) observed ASVs, (B) Faith’s phylogenetic diversity, and (C) Pielou’s evenness. Between-condition differences were determined by linear mixed models comparing pistachio and usual care conditions adjusting for precondition values. Averages were calculated with a custom R function that randomly subsampled the abundance table (rrarefy) and calculated *⍺-*diversity metrics after each subsampling of the raw values (repeated *n* = 100 times). The usual care condition refers to education to consume 1–2 carbohydrate exchanges (15–30 g carbohydrates) as a nighttime snack. ASVs, amplicon sequence variants.

A total of 19 samples were omitted from diversity analyses after filtering samples to a minimum depth of 10,000 reads [*n* = 12 from the pistachio condition (9 precondition); *n* = 7 usual care condition (4 precondition)]. The change in relative abundance from precondition values of bacterial phyla and the most abundant genera or species are depicted in Figure 1. Bacteriodota, Firmicutes, and Actinobacteria were among the most abundant phyla at each timepoint. No significant between-condition differences were observed for α-diversity (Table 2) assessed by number of observed ASVs (Figure 2A), Faith’s Phylogenetic Diversity (Figure 2B), Pielou’s Evenness (Figure 2C), Shannon Index (Figure 2D), Simpson Index (Figure 2E), or Chao-1 Index (Figure 2F).

**TABLE 2:** Mean differences in stool microbial community ⍺-diversity between the usual care and pistachio conditions among individuals with prediabetes.

⍺-diversitymetric	Mean difference(usual care vs. pistachio)	95% confidenceinterval
Observed ASVs	2.06	–7.15, 11.14
Faith’s PD	0.07	–0.47, 0.61
Pielou’s Evenness	–0.01	–0.04, 0.02
Shannon Index	–0.04	–0.20, 0.12
Simpson Index	–0.01	–0.03, 0.02
Chao-1 Index	3.11	–7.19, 13.1

Abbreviations: ASVs, amplicon sequence variants; PD, phylogenetic diversity.

Data were tested using linear mixed models comparing post usual care vs. post pistachio conditions adjusting for precondition values. Usual care is defined as education to consume 1–2 carbohydrate (CHO) exchanges each night (15–30 g CHO).

### β-Diversity

**FIGURE 3:**
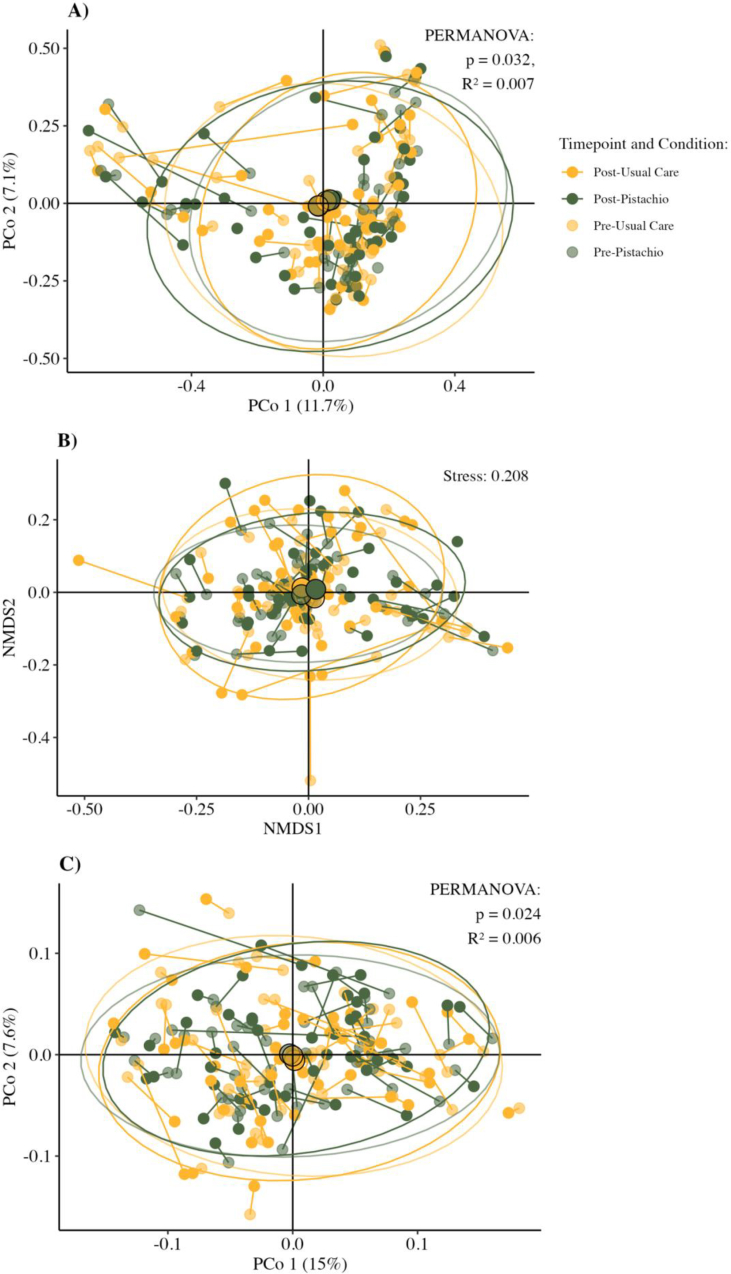
Metric and nonmetric multidimensional scaling of microbial community β-diversity. (A) Principal coordinate analysis (PCoA) ordination of Bray–Curtis dissimilarity by timepoint and condition. Differences in community diversity between samples at each timepoint were tested by permutational analysis of variance (PERMANOVA) using the Adonis2 package and stratified by participant. A large degree of variance in community diversity was explained by including participant as a covariate in the model (*R*^2^ = 0.80, *P* = 0.23). (B) Nonmetric multidimensional scaling of Bray–Curtis dissimilarity by timepoint and condition. (C) PCoA ordination of weighted Unifrac distance colored by timepoint and condition. PCoA, principal coordinate analysis; PERMANOVA, permutational analysis of variance.

β-diversity metrics clustered by timepoint and condition are depicted in Figure 3A–C. For Bray–Curtis dissimilarity, a significant effect was observed when comparing timepoints and conditions (PERMANOVA, *P =* 0.032) (Figure 3A). Pairwise PERMANOVA showed community dissimilarity between the pistachio and usual care conditions at the postcondition timepoint (*P =* 0.001) (Table 3). Little variation in community diversity was explained by study condition (*R*^2^ = 0.007). Weighted Unifrac dissimilarity metrics also identified significant clustering (PERMANOVA, *P =* 0.024) (Figure 3C). Differences were observed between conditions (*P =* 0.012; *R*^2^ = 0.007), within the pistachio condition (*P =* 0.007; *R*^2^ = 0.006), and between pre-usual care and post-pistachio (*P =* 0.034, *R*^2^ = 0.005).

**TABLE 3:** Pairwise permutational analysis of variance results for dissimilarity metrics by timepoint and condition in adults with prediabetes.

Comparison	F-statistic	*p* value	*R*^2^
Bray–Curtis dissimilarity			
Pre-usual care vs. pre-pistachio	0.401	0.277	0.005
Pre-usual care vs. post-usual care	0.274	0.612	0.003
Pre-usual care vs. post-pistachio	0.358	0.187	0.004
Pre-pistachio vs. post-usual care	0.436	0.289	0.005
Pre-pistachio vs. post-pistachio	0.486	0.066	0.006
Post-pistachio vs. post-usual care	0.615	0.001	0.007
Weighted Unifrac dissimilarity			
Pre-usual care vs. pre-pistachio	0.296	0.403	0.003
Pre-usual care vs. post-usual care	0.204	0.647	0.002
Pre-usual care vs. post-pistachio	0.521	0.034	0.005
Pre-pistachio vs. post-usual care	0.289	0.429	0.003
Pre-pistachio vs. post-pistachio	0.557	0.007	0.006
Post-pistachio vs. post-usual care	0.687	0.012	0.007

Data were tested using pairwise permutational analysis of variance (pairwise.adonis2) comparing all timepoints and conditions. *P* values are adjusted using Benjamini–Hochberg correction. Usual care is defined as education to consume 1–2 carbohydrate (CHO) exchanges each night (15–30 g CHO).

**FIGURE 4:**
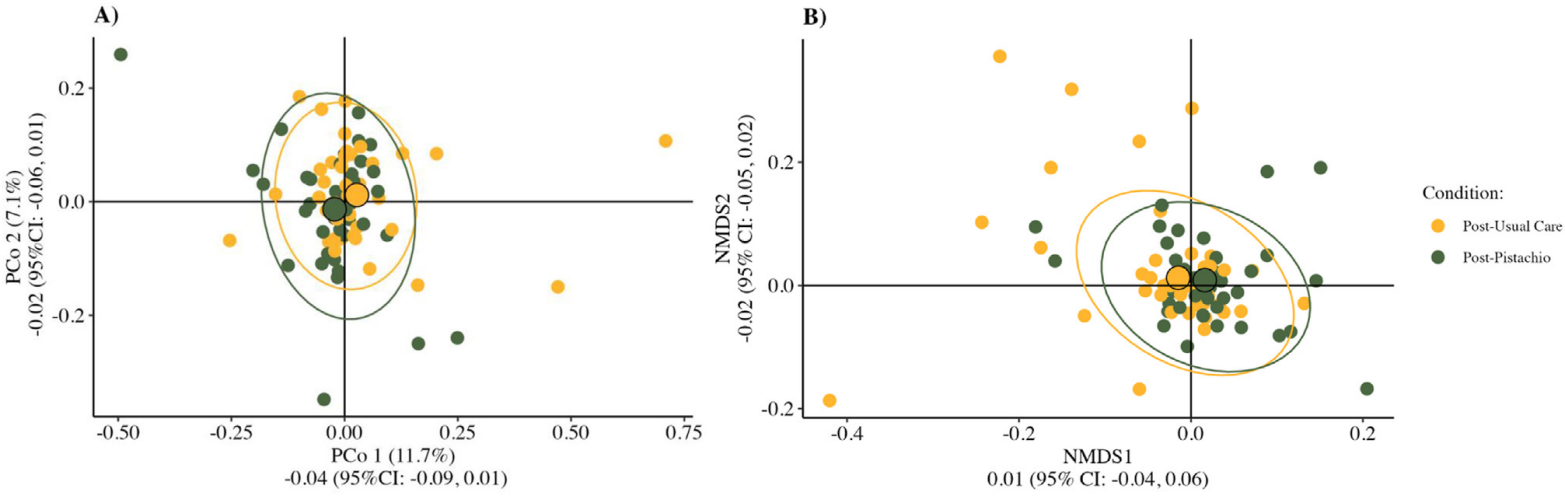
Baseline-centered metric and nonmetric multidimensional scaling of microbial community β-diversity. *(*A) Principal coordinate analysis (PCoA) ordination of Bray–Curtis dissimilarity postcondition after adjusting for distance from precondition for each participant. (B) Nonmetric multidimensional scaling analysis plot depicting Bray–Curtis dissimilarity postcondition after adjusting for distance from precondition for each participant. Both plots illustrate the comparison of β-diversity distance metrics by axis using linear mixed models adjusting for precondition distances. Results for each axis are reported as least-squares mean differences and [95% confidence intervals (CIs)]. NMDS1 includes the interaction term condition by randomization sequence in the final model. NMDS, nonmetric dimensional scaling.

Because distance measures for Bray–Curtis dissimilarity tended to cluster by participant (*R*^2^ = 0.80; Supplemental Figure 1), we attempted to reduce variance by testing differences between conditions by axis for principal coordinate analysis (PCoA) and NMDS, which is depicted in Figure 4A and B. There were no differences in dissimilarity distance measures by condition for either PCoA axes (Figure 4A). When testing differences in distances along NMDS axes, a condition by randomization sequence interaction was observed, which is indicative of carryover effects (Figure 4B). No differences were observed for NMDS2.

### Taxa abundance

**FIGURE 5:**
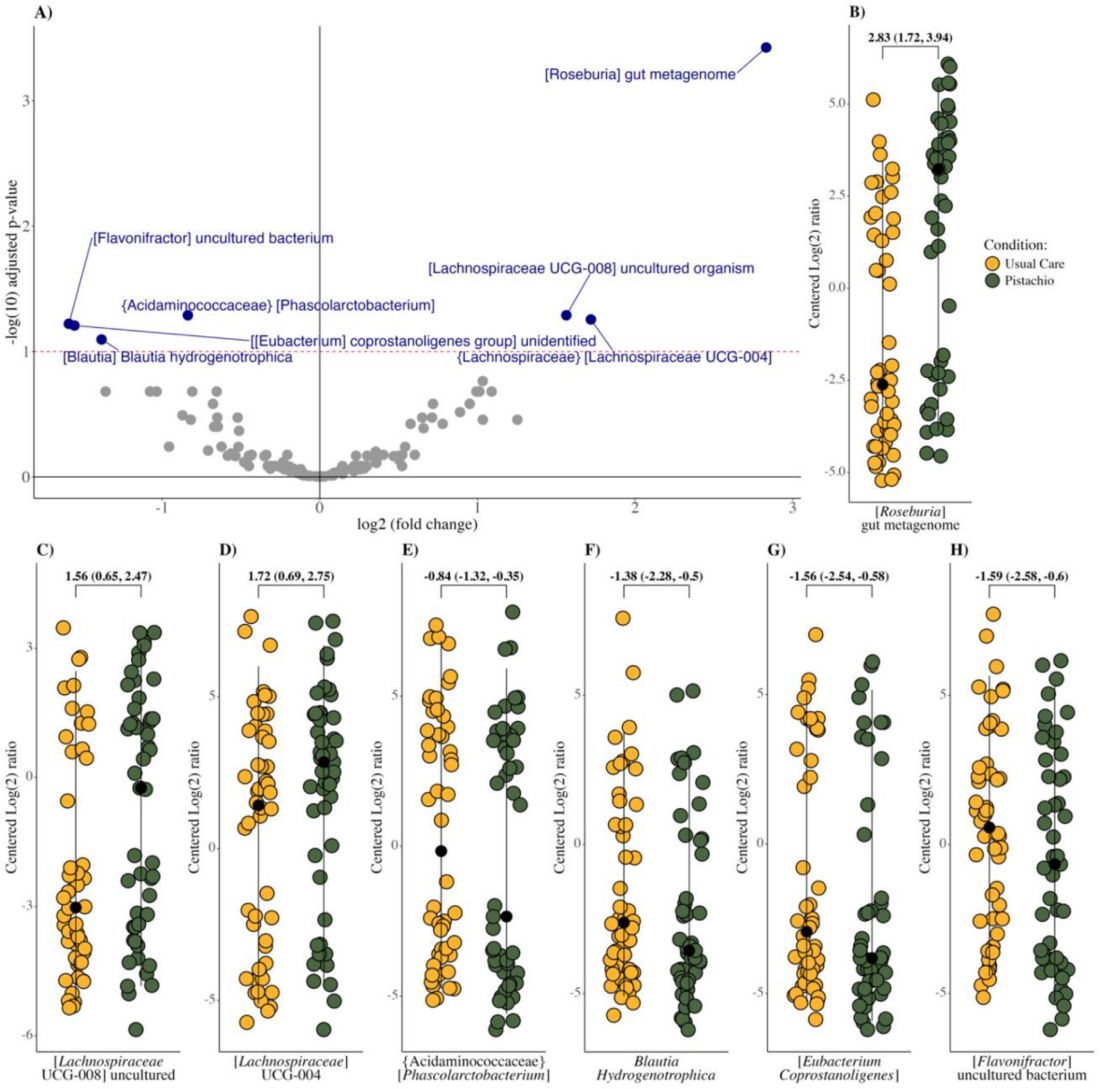
Postcondition mean differences in microbial taxa among adults with prediabetes. Postcondition differences were tested using linear mixed models adjusting for precondition taxa abundance. Comparisons were made after raw counts were transformed using centered log(2)-ratio (count zero multiplicative). (A) Volcano plot of significant microbial taxa (log2 – fold difference) by –log(10) adjusted *P* values. The red line indicates the level of significance with Benjamini-Hochberg correction P < 0.1. (B–H) Significant microbial taxa when comparing post-usual care compared with post-pistachio conditions. Taxa are depicted on the centered log(2) ratio scale. Results are reported as mean difference (95% confidence intervals). “{}” refers to family name and “[]” refers to genus name.

Several microbial taxa within the Firmicutes phylum were altered when comparing conditions after Benjamini–Hochberg correction of *P* values (*P* < 0.0058 ≈ FDR_p = 0.1; 130 microbial taxa in the Benjamini-Hochberg correction) (Figure 5A–H). Compared with usual care, nighttime pistachio consumption enriched the *Roseburia* metagenome (log(2)-fold mean difference: 2.83; 95% CI: 1.72, 3.49), and *Lachnospiraceae* uncultured genera (UCG) –004 (1.72; 95% CI: 0.69, 2.75) and −008 (1.56; 95% CI: 0.65, 2.47). Microbial taxa that decreased when comparing usual care with the pistachio condition included the genus *Flavonifractor* (–1.59; 95% CI: –2.58, –0.6), [*Eubacterium*] *coprostanoligenes* group (–1.56; 95% CI: –2.54, –0.58), *Phascolarctobacterium* (–0.84; 95% CI: –1.32, –0.35), and *Blautia hydrogenotrophica* (–1.38; 95% CI: –2.28, –0.5). No other taxa were significantly different between conditions.

We then explored whether differentially abundant microbial taxa were associated with differences in diet quality scores because the HEI-2020 score was higher (5.51 points; 95% CI: 0.79, 10.28) after the pistachio condition compared with the usual diet condition (Supplemental Table 2). The change in HEI-2020 scores was not associated with the abundance of microbial taxa that were modified by treatment (data not reported).

## Discussion

In this study, we evaluated differences in microbial composition from stool samples of adults with prediabetes after the consumption of 57 g of pistachios as a nighttime snack for 12-wk compared with education to consume 1–2 CHO exchanges (15–30 g CHO). There was a small yet significant dissimilarity in bacterial community diversity between stool samples after consumption of pistachios as a nighttime snack compared with education to consume 15–30 g CHO as a nighttime snack. The abundance of the microbial features *Roseburia* metagenome, *Lachnospiraceae* UCG-004, and UCG-008 was greater with pistachio consumption whereas *Flavonifractor*, *Eubacterium coprostanoligenes* group, *Phascolarctobacterium*, and *B. hydrogenotrophica* were less abundant compared with the usual care condition. There were no differences in α-diversity between conditions. These findings suggest that intake of 57 g/d of pistachios, compared with higher CHO evening snacks, for 12 wk produces small differences in the microbial community of stool samples from adults with prediabetes without affecting microbial richness and evenness.

Nighttime pistachio consumption resulted in dissimilar bacterial community diversity compared with the usual care condition. These differences in community diversity profiles occurred with a dietary intervention that reflects clinical management for elevated fasting glucose. However, testing community diversity by NMDS axis identified a carryover effect. Ukhanova et al. [7] found that β-diversity was unaffected by consumption of fully controlled diets with 42 or 85 g/d of pistachios compared with a no nut diet for 18 d in generally healthy adults. Despite controlling all foods, fiber intakes were slightly higher with pistachio-enriched diets [control = 32.7 g/d fiber, pistachios (42 g/d) = 35.2 g/d, pistachios (85 g/d) = 37.6 g/d], which is expected to increase the availability of undigested foodstuffs to the gut microbiota. Our trial observed similar differences in fiber intake after the pistachio condition (+5.0 g; 95% CI: 1.1 g, 8.8 g) but cannot fully attribute the effects to pistachio consumption because other foods were not controlled. To the extent that pistachios influenced stool bacterial community diversity, the fiber content is not expected to have direct microbial effects. The predominant fiber in pistachios is insoluble [5], which is poorly fermented by gut bacteria [39]. It is possible that the insoluble fiber in pistachios may indirectly affect community diversity by increasing food transit time and limiting bacterial digestion of nondigested foodstuff [39]. The transit time of food through the gut has been closely associated with changes in stool microbiota and lipid and glucose responses [40]. Whether insoluble fibers from pistachios alter gut transit time is not well understood.

Similar to previous trials examining nut intake, we observed higher abundance of metagenomic features associated with *Roseburia* after pistachio consumption. Microbial features in the genus *Roseburia* are major butyrate-producing bacteria [41]. Butyrate is a primary fuel source for colonocytes providing ∼70% of their energy requirements [42]. Butyrate production from the microbial community is also associated with cell barrier function, cell turnover, and reduced inflammation, which support a healthy commensal relationship between the gut microbiota and the human host [41]. The enrichment of the *Roseburia* metagenome after the pistachio condition suggests that even small dietary changes such as consumption of pistachios instead of CHOs favors gut health potentially through short-chain fatty acid (SCFA) production. This effect may be representative of nut intake in general as the enrichment of *Roseburia* has been observed in other studies with peanut and tree nut interventions. Choo et al. [43] observed higher relative abundance of *Roseburia* with 56 g/d of almonds for 8 wk in adults with elevated fasting glucose compared with a higher CHO snack*.* A higher relative abundance of *Roseburia* has also been shown after peanut consumption (27 g/d) compared with a lower-fat higher CHO nighttime snack over 6 wk [44]. Holscher et al. [45] observed a higher relative abundance of *Roseburia* (*P* < 0.05) with almond consumption (42 g/d) compared with no almonds for 3 wk. The enrichment of *Roseburia* with tree nuts or peanut consumption is important considering that nuts are recommended as part of healthful diets [2]. Given this, more work is needed to understand the functional implications of *Roseburia* enrichment with nut intake.

Several other microbial taxa were altered in response to nighttime pistachio consumption. Pistachio consumption resulted in greater abundance of microbiota in the *Lachnospiraceae* family (uncultured groups 004, 008, *Roseburia* metagenome). The *Lachnospiraceae* family of bacteria encompasses a diverse group of SCFA producers. Although butyrate is the preferred SCFA for colonocytes, propionate, and acetate also contribute to a healthful microbial community and activate the gut brain axis. *B. hydrogenotrophica* is also a member of the *Lachnospiraceae* family but was reduced with nighttime pistachio consumption compared with the usual care condition. *B. hydrogenotrophica* is a producer of aromatic amino acids (precresyl sulfate) that act as precursors to uremic toxins [46]. Stool microbial effects of pistachio consumption may also be reflected by a lower abundance of *Eubacterium flavonifractor*, which degrades dietary flavonoids [47]. Flavonoid metabolites have a wide array of metabolic functions, although, the metabolic implications remain unclear [48]. Pistachio consumption was also associated with lower abundance of *E. coprostanoligenes* and *Phascolarctobacterium* genera, which have been linked to gut barrier function [49] and acetate/proprionate production [50], respectively. Although pistachio consumption influenced the abundance of several microbial taxa that are associated with metabolic health, our primary analysis did not reveal significant differences in glycemic control, vascular health, or lipids/lipoproteins [14], suggesting that microbial changes in response to this low-intensity intervention were independent of metabolic effects.

Interpretation of these findings should be considered in the context of the strengths and limitations of the study. The strengths of this study are the crossover trial design, high adherence to the dietary protocol, and use of 24-h recalls for dietary assessment. The randomized crossover clinical trial design allowed for assessment of between-condition differences while accounting for within-person differences to reduce variance in the data. Adherence was very good with an average adherence of 91%. The limitations of this study include the lack of dietary control, which could contribute to microbiota effects and the reported higher fiber intake after the pistachio condition, which may explain differences in the microbiota. Other bioactive components in pistachios (i.e., phenolic compounds) may also drive the microbial effects but were not identified through analysis of pistachios provided in this trial. 16S rRNA gene sequencing has limitations, such as the inability to distinguish between closely related species and directly assess functional potential within microbial communities. To gain deeper insights into the complex interactions between diet and the microbiome, future studies could incorporate additional methodologies, including metagenomic sequencing, metabolomic approaches, or experimental validation to demonstrate causality of microbial shifts driving host metabolic phenotypes. Combined with our analyses, these methods would ultimately enhance our understanding of the microbiome’s response to dietary interventions. Finally, this study used LMMs for testing, which is consistent with the parent trial and recommended for analysis of crossover designs. The absence of carryover is a fundamental assumption with crossover trial designs and, although carryover effects were not determined to influence most tests, this study is likely underpowered to detect carryover effects. Limited statistical power is also a concern for differential abundance testing because most findings were of moderate-to-large effect sizes.

In conclusion, we observed clustering of gut microbial communities after pistachio intake that were driven by a greater abundance of *Roseburia metagenome* and *Lachnospiraceae* and a lower abundance of *Flavonifractor*, *Eubacterium*, *Phascolarctobacterium*, and *Blautia* genera. These findings suggest that intake of 57 g/d of pistachios as a nighttime snack, compared with higher CHO evening snacks, for 12 wk alters microbiota composition in adults with prediabetes. Whether pistachios influence the functional potential of the gut microbiota requires further investigation.

## Author contributions

The authors’ responsibilities were as follows – KSP, PMK-E, RL: designed the research; TMR, KK, RL: conducted the research; TMR, KSP, JW, JCS, KK, JEB: contributed to analysis and interpretation; TMR: drafted the manuscript; PMK-E, RL, KK, JCS, KSP, JW, JEB: critically reviewed the manuscript; KSP: had primary responsibility for final content; and all authors: read and approved the final manuscript.

## Funding

The American Pistachio Growers; Penn State’s Clinical & Translational Research Institute, Pennsylvania State University CTSA, NIH/NCATS Grant Number 1UL1TR002014-01. This research was also supported by a grant to Juniata College from the Howard Hughes Medical Institute (http://www.hnmi.org) through the Precollege and Undergraduate Science Education Program, as well as by the National Science Foundation (http://www.nsf.gov) through NSF award DBI-1248096. The content is solely the responsibility of the authors and does not necessarily represent the official views of the NIH.

## Conflict of interest

KSP and PMK-E received a grant to conduct the research from The American Pistachio Growers. JW and RL are cofounders of Wright Labs LLC. The other authors report no conflicts of interest.
