# Supplementary material for: Nighttime Pistachio Consumption Alters Stool Microbiota Diversity and Taxa Abundance Compared with Education to Consume 1–2 Carbohydrate Exchanges (15–30 grams) over 12 Weeks in Adults with Prediabetes: A Secondary Analysis from a Randomized Crossover Trial
Source: Curr Dev Nutr. 2025 May 27;9(7):107481. doi: 10.1016/j.cdnut.2025.107481 (PMC12221629; doi:10.1016/j.cdnut.2025.107481)
Supplement: multimedia component 1 [file mmc1.docx]

# Supplementary materials

Nighttime pistachio consumption alters stool microbiota diversity and taxa abundance compared to education to consume 1-2 carbohydrate exchanges (15-30 g) over 12 weeks in adults with prediabetes: a secondary analysis from a randomized crossover trial.

Terrence M. Riley

Justin Wright

Gina Lamendella

Jordan Bisanz

Khushi Kanani

Jeremy Chen See

Penny Kris-Etherton

Kristina Petersen

# Table S1 Daily nutrient intake among study completers (n = 51)

| Nutrient | Pistachio | |  | Usual care | |
| --- | --- | --- | --- | --- | --- |
|  | Pre (n = 49) | Post (n = 42) |  | Pre (n = 44) | Post (n = 45) |
| Energy (kcal) | 2131 (772) | 2157 (738) |  | 2316 (922) | 2140 (836) |
| Protein (g) | 93.7 (34.1) | 100.1 (43.9) |  | 104.4 (51.9) | 96.5 (45.8) |
| Protein (%) | 18.3 (6) | 18.5 (4.8) |  | 18 (5.7) | 18.7 (7.1) |
| CHO (g) | 232.1 (104.2) | 217 (86.9) |  | 239.9 (87.2) | 229.8 (105.2) |
| CHO (%) | 43.6 (11.8) | 40.7 (10.4) |  | 43.1 (11.3) | 43.8 (13.1) |
| Fat (g) | 91.1 (42.1) | 98.8 (41.7) |  | 102.9 (58.8) | 89.7 (52.5) |
| Fat (%) | 38 (10.3) | 41.1 (9.4) |  | 38.8 (9.4) | 36.9 (9.8) |
| SFA (g) | 29.8 (15) | 29.4 (14.1) |  | 37.2 (25.6) | 29.5 (21) |
| SFA (%) | 12.5 (4.7) | 12.4 (4.3) |  | 13.9 (4.9) | 12.1 (4.4) |
| MUFA (g) | 32.8 (17.8) | 37.6 (20) |  | 36.3 (22.7) | 32.3 (22.4) |
| MUFA (%) | 13.6 (4.8) | 15.3 (4.3) |  | 13.4 (4) | 12.9 (4.2) |
| PUFA (g) | 20.6 (11.8) | 23.4 (11.3) |  | 20.6 (9.7) | 20.4 (12.1) |
| PUFA (%) | 8.6 (3.6) | 9.8 (3.9) |  | 8.1 (2.7) | 8.7 (4) |
| Fiber (g) | 19.9 (10.6) | 22.4 (13.2) |  | 21.2 (10.3) | 19 (11.1) |
| Potassium (mg) | 3042 (1274) | 3117 (1405) |  | 3290 (1545) | 2814 (1101) |
| Sodium (mg) | 3682 (1598) | 2157 (738) |  | 4312 (1695) | 2140 (836) |

Data are reported from 24-hr dietary recalls using ASA24. Usual care is defined as education to consume 1–2 carbohydrate (CHO) exchanges (15–30 g CHOs) before bedtime but after dinner.

Values are arithmetic mean ± SD.

# Figure S1 Clustering of Bray-Curtis dissimilarity by participant in metric dimensional scaling (PCoA)


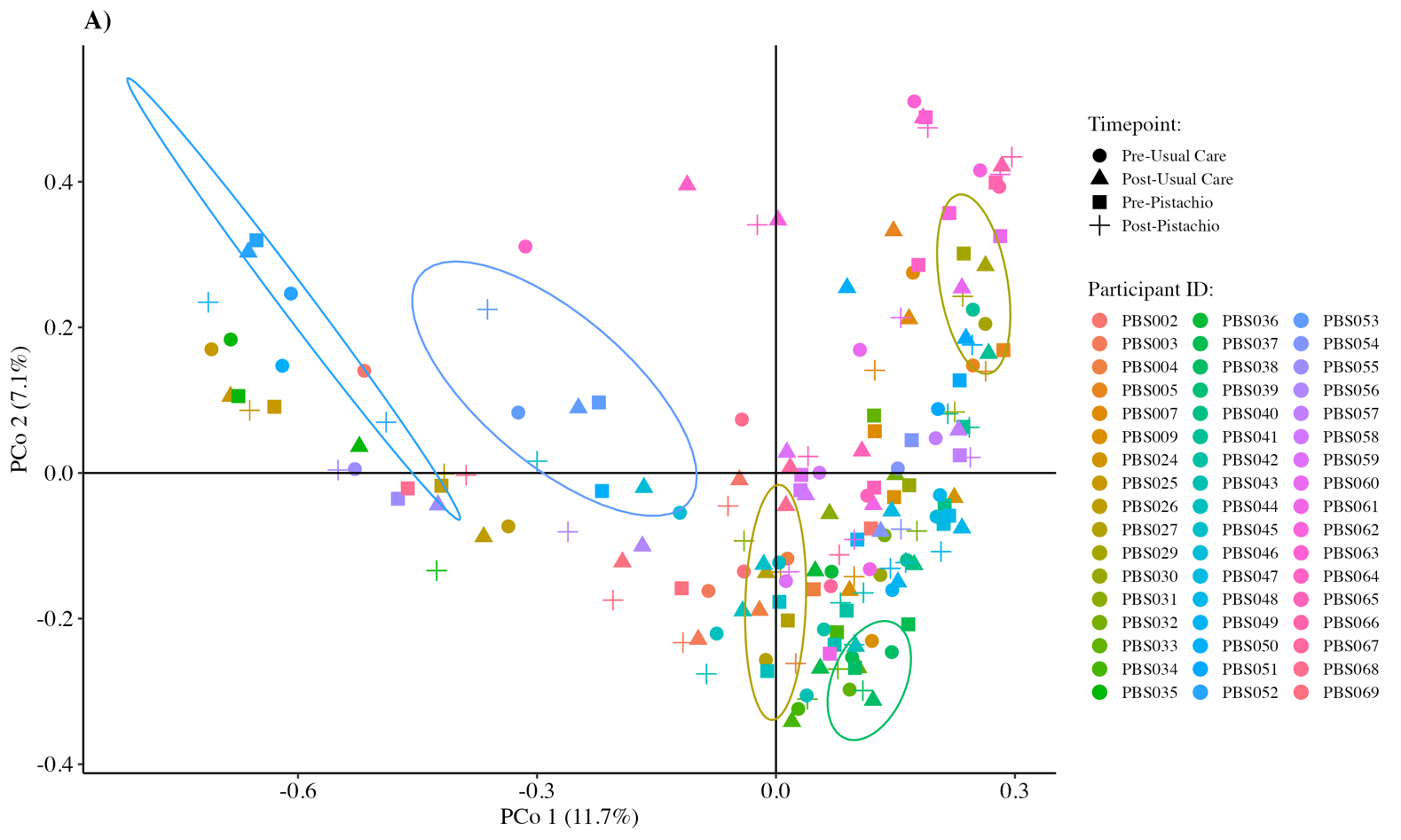


PCoA of Bray-Curtis dissimilarity for each timepoint and condition colored by participant. Microbial communities between samples cluster by participant as depicted by ellipses for selected participants. Shapes indicate the timepoints and conditions and ellipses indicate 90% confidence interval. PCoA, principal coordinate analysis

# Table S2 Differences in glycemic indicators and diet quality between conditions for individuals with prediabetes

| Characteristic | Mean difference | 95% Confidence Interval | |
| --- | --- | --- | --- |
| FPG (mg/dL) | 0.81 | -1.04 | 2.64 |
| Insulin (µU/mL) | 0.90 | -0.50 | 2.30 |
| HOMA-IR | 0.48 | -0.01 | 0.98 |
| HbA1c (%) | 0.01 | -0.04 | 0.06 |
| **HEI-2020 (points)** | **5.51** | **0.80** | **10.28** |

Differences were determined by linear mixed models including base values as fixed effects and participants as random effects. FPG, fasting plasma glucose, HbA1c, glycated hemoglobin; HEI-2020, healthy eating index-2020; HOMA-IR, homeostatic model assessment of insulin. resistance.

# Custom R code for ⍺-diversity metrics:

Input data is a data frame with samples as rows and taxa as columns

**Observed ASVs:**

ObFeats_avg = function() {

standard_depth %>% # *Uses the input table after removing samples with < 10K reads*

rrarefy(., sample = minStandard) %>% *# Rarefy to 10K reads*

specnumber()} *# Calculate the number of observed species with > 0 reads per sample (richness)*

set.seed(63063) *# Setting the seed for reproducibility*

observed_features = replicate(100, ObFeats_avg()) %>% *# Run observed_features function 100x returning a matrix rows as sampleIDs and columns as the replicate number*

as_tibble(rownames = "sampleID", .name_repair = "unique") %>% *# Convert the matrix to a tibble without duplicating column names (.name_repair) and move rownames to a column with an identifier consistent across tables*

pivot_longer(-sampleID) %>% *# convert the tibble object to the long format*

group_by(sampleID) %>% *# Group by sampleID to prepare the average calculation*

summarize(Observed = mean(value)) *# Create a new long table with the average of FPD for each person at each timepoint*

**Faiths phylogenetic diversity:**

FPD_avg = function() {

standard_depth %>% # *Uses the input table after removing samples with < 10K reads*

rrarefy(., sample = minStandard) %>% *# Rarefy to 10K reads*

pd(., tree, include.root = T) %>% *# Calculates phylogenetic diversity*

as.data.frame() %>% *# Convert to a data frame*

rownames_to_column(var = "sampleID") %>% *# Move rownames to a column with an identifier consistent across tables*

select(!SR) *# Remove species richness from since we only want phylogenetic diversity*

set.seed(63063) *# Setting the seed for reproducibility*

FPD = replicate(100, FPD_avg(), simplify = F) %>% *# Run FPD_avg function 100x returning 100 data frames as a list (simplify = F)*

map_dfr(., ~ .x) *# Creates a long data frame with results from 100x FPD by sampleID (each individual at each timepoint)*

FPD_avg = FPD %>% *# Create a new data frame for the average FPD*

group_by(sampleID) %>% *# Group by sampleID to prepare the average calculation*

summarize(FPD = mean(PD)) *# Create a long table with the average of FPD for each person at each timepoint.*

**Shannon diversity and Pielou’s evenness index:**

shannon_avg = function() {

standard_depth %>% # *Uses the input table after removing samples with < 10K reads*

rrarefy(., minStandard) %>% *# Rarefy to 10K reads*

diversity(., index = "shannon")} *# Calculates Shannon diversity needed for Pielou’s evenness*

set.seed(63063) *# Setting the seed for reproducibility*

Shannon = replicate(100, shannon_avg()) %>% *# Run shannon_avg function 100x returning a matrix with rows as sampleIDs and columns as the replicate number*

as_tibble(rownames = "sampleID", .name_repair = "unique") %>% # *Convert the matrix to a tibble without duplicating column names (.name_repair) and move rownames to a column with an identifier consistent across tables*

pivot_longer(-sampleID) %>% *# Convert the tibble object to the long format*

group_by(sampleID) %>% *# Group by sampleID to prepare the average calculation*

summarize(Shannon = mean(value)) *# Create a long table with the average of Shannon index for each person at each timepoint*

Pileou = Shannon %>% *# Create a new data frame for the calculation of evenness based on average Shannon diversity*

dplyr::select(., sampleID=`(sampleID)`, shannon=`(Shannon = mean(value))`) %>% *# Modify column names*

mutate(pielou = shannon/log(specnumber(standard_depth))) *# Calculate Pielou’s evenness*

**Shannon diversity index:**

simpson_avg = function(){

standard_depth %>% # *Uses the input table after removing samples with < 10K reads*

rrarefy(., minStandard) %>% *# Rarefy to 10K reads*

diversity(., index = "simpson")} *# Calculates Simpson diversity index*

set.seed(63063) *# Setting the seed for reproducibility*

Simpson = replicate(100, simpson_avg()) %>% *# Run simpson_avg function 100x returning a matrix with rows as sampleIDs and columns as the replicate number*

As_tibble(rownames = "sampleID", .name_repair = "unique") %>% # *Convert the matrix to a tibble without duplicating column names (.name_repair) and move rownames to a column with an identifier consistent across tables*

pivot_longer(-sampleID) %>% *# Convert the tibble object to the long format*

group_by(sampleID) %>% *# Group by sampleID to prepare the average calculation*

summarize(Simpson = mean(value)) *# Create a long table with the average of Simpson index for each person at each timepoint*

**Chao-1 index:**

sample_chao = function(data){

chao_index = apply(data, 1, function(counts){ *# Uses apply function to go down the rows (each sample) and…*

single = sum(counts == 1) *# Counts how many taxa have exactly 1 read*

double = sum(counts == 2) *# Counts how many taxa have exactly 2 reads*

S_obs = sum(counts > 0) *# Calculate the number of observed species with > 0 reads per sample (richness)*

if(double == 0){ *# Checks if no taxa have only 2 counts*

chao1 = S_obs + (single * (single - 1)) / (2 * (double + 1))} *# Bias corrected chao-1 calculation*

else { *# If there are taxa with 2 count then use classic Chao-1 formula*

chao1 = S_obs + (single^2) / (2 * double)} *# Classic Chao-1 formula*

return(chao1)}) *# Return Chao-1 corrected or*

return(chao_index)} *# Return Chao-1*

chao_avg = function() {

rarefied = rrarefy(standard_depth, sample = minStandard) # *Uses the input table after removing samples with < 10K reads to rarefy to 10K reads for all samples*

sample_chao(rarefied)} # *Calculates chao-1 index for each sample*

set.seed(63063) *# Setting the seed for reproducibility*

Chao1_avg = replicate(100, sample_chao_avg()) %>% *# Run chao_avg function 100x returning a matrix with rows as sampleIDs and columns as the replicate number*

as.data.frame() %>% *# Convert to data frame*

rownames_to_column(var = "sampleID") %>% # *Move rownames to a column with an identifier consistent across tables*

pivot_longer(-sampleID, values_to = "chao") %>% *# Convert the data frame object to the long format*

group_by(sampleID) %>% *# Group by sampleID to prepare the average calculation*

summarize(chao = mean(chao)) *# Create a long table with the average of Chao-1 index for each person at each timepoint*
